# Hyponatremia – predictor of adverse prognosis in cirrhosis

**Published:** 2012-06-18

**Authors:** A Bengus, RD Babiuc

**Affiliations:** Gastroenterology Department, University Emergency Hospital, Bucharest

**Keywords:** hyponatremia, hypo-osmolarity, circulatory dysfunction, hepatorenal syndrome

## Abstract

Hyponatremia is a frequent complication of the advanced liver disease, being, as the hepatorenal syndrome, a consequence of the important circulatory dysfunction of cirrhosis. Hyponatremia is determined by the impaired capacity of the kidney to excrete free water, which leads to water retention disproportionate to sodium retention, thus causing low plasma osmolarity. Hyponatremia in cirrhosis is associated with a high morbidity and mortality, its presence suggesting a very advanced liver disease. Current evidence suggests that hyponatremia affects the brain function and predisposes to hepatic encephalopathy. In addition, hyponatremia is a risk factor for liver transplantation, being associated with a high frequency of complication and affecting short and long-term post-transplant survival.

Cirrhosis is characterized by a progressive circulatory dysfunction, including systemic arterial vasodilatation and reduced peripheral resistance, which induces renal hypoperfusion. Renal hypoperfusion represents the stimulus that activates the renin angiotensin aldosteron system having as consequence sodium and water retention [**1**]. 

Hyponatremia in cirrhosis was first described in the 1950′s, but its importance was overlooked for many years. 30 years later its importance as a predictor for the survival in cirrhosis was investigated [**2,3**].

## Definitions

Currently, hyponatremia in cirrhosis is defined as having a serum sodium level below 130 mmol/l [**4**]. According to this definition, the prevalence of hyponatremia in cirrhotic patients is of about 21,6%. If the cut-off limit for serum sodium is considered to be of 135 mmol/l (that represents the lower limit of serum sodium in healthy subjects), then the prevalence is of about 49,4% [**5**].

Cirrhotic hyponatremia is associated with jaundice, hepatic encephalopathy, refractory ascites and hepatorenal syndrome [**5**]. Serum sodium in cirrhosis that is below 130 mmol/l is associated with a median transplant-free survival of less than 6 months [**6**]. 

2 types of hyponatremia have been described in cirrhotic patients:

- Hypovolemic hyponatremia – this condition is due to important losses of extracellular fluids (excess of diuretics or losses in the gastrointestinal tract); it is characterized by low serum sodium and low plasma volume. The patients do not have ascites or edema; they have signs of dehydration and prerenal azotemia.

- Hypervolemic hyponatremia – also named dilutional hyponatremia. This condition is associated with large ascites (frequently refractory ascites) and edema. It is caused by the renal impairment in excretion of solute-free water, causing disproportionate water retention compared to sodium. Therefore, in this case, the plasma volume is expanded in absolute value, but it is low compared to the marked arterial dilatation characteristic to advanced cirrhosis.

Because most of the time hyponatremia characterizes advanced cirrhosis (when the patients also have many other derangements), it is difficult to identify the clinical manifestations induced by hyponatremia per se.

Hepatic encephalopathy is the most important clinical complication of hyponatremia. Besides this, hyponatremia is associated with other complications of cirrhosis. In the majority of patients, it occurs together with acute kidney injury or even HRS, and thus, correlates with a poor prognosis. It is important to know that cirrhotic patients with hyponatremia are at very high risk of developing the hepatorenal syndrome [**7**]. In this situation, probably, hyponatremia is due to increased levels of arginine vasopressine (HRS is characterized by intense stimulation of the renin angiotensin aldosterone system due to an extreme systemic vasodilatation) and to reduced glomerular filtration rate and increased proximal tubular sodium reabsorption [**8**].

Cirrhotic patients with hyponatremia are found more frequently in cases of bacterial infections [**9**].

Several lines of evidence support the existence of a correlation between hyponatremia and hepatic encephalopathy. Levels of serum sodium and ammonia determine the major electroencephalographic changes in cirrhosis [**10**]. The novel theories suggest that low-grade cerebral edema (which can be induced by hyponatremia) may play a part in the pathogenesis of hepatic encephalopathy [**11**]. This low-grade cerebral edema, resulting from the swelling of the astrocytes (maybe by increased intracellular content of glutamine, resulted from ammonia metabolism) is responsible for a number of alterations of the neurological functions, which can lead to hepatic encephalopathy. In this context of the existence of low-grade cerebral edema, hyponatremia plays an important role in increasing the osmotic pressure on the astrocytes. In this situation, only small increases in ammonia levels can induce clinically manifested hepatic encephalopathy.

After liver transplantation, once the important circulatory dysfunction induced by hepatic failure is removed, serum sodium levels improves rapidly, however, the risk of central pontine myelinolysis in the early postoperative period still exists [**12,13**].

The impact of serum sodium on the prognosis in patients with advanced liver disease was evaluated in a study that included over 6000 patients, candidates for liver transplantation in the United States [**1**]. The hazard rate for death was of 1.05 per 1 mmol/l decrease in serum sodium at values between 140 and 125 mmol/l.

2 studies [**15,16**] suggested that pretransplant hyponatremia is associated with a high risk of neurological complications, of renal failure and infectious complications, longer hospitalization and with an increased short-term mortality rate after transplantation. Another study [**17**] showed that among cirrhotic patients with normal serum sodium that underwent liver transplantation, those with a history of hyponatremia in the preceding 6 months had poorer outcomes. Thus, the correction of serum sodium seems not to eliminate the associated mortality risk.

In addition, cirrhosis hyponatremia affects the quality of life of the patients because they require a fluid intake restriction in order to prevent further dilution, and is usually not very well tolerated. In a recent study [**18**], hyponatremia was an independent predictive factor of the altered quality of life in a patient with cirrhosis.

However, data available until now suggest that hyponatremia in cirrhosis is only a manifestation of the profound circulatory dysfunction, and that correction of hyponatremia per se has only limited benefits if the underlying circulatory condition is not improved.
